# Color Affects Recognition of Emoticon Expressions

**DOI:** 10.1177/20416695221080778

**Published:** 2022-02-28

**Authors:** Songyang Liao, Katsuaki Sakata, Galina V. Paramei

**Affiliations:** Graduate School of Human Sciences, 12853 Kanagawa University, Kanagawa, Japan; Department of Fine Arts, 12841 Joshibi University of Art and Design, Tokyo, Japan; Department of Psychology, Liverpool Hope University, UK

**Keywords:** emoticons, decoding emotion expressions, color, affective meaning, affective congruency, Japan

## Abstract

In computer-mediated communication, emoticons are conventionally rendered in yellow. Previous studies demonstrated that colors evoke certain affective meanings, and face color modulates perceived emotion. We investigated whether color variation affects the recognition of emoticon expressions. Japanese participants were presented with emoticons depicting four basic emotions (Happy, Sad, Angry, Surprised) and a Neutral expression, each rendered in eight colors. Four conditions (E1–E4) were employed in the lab-based experiment; E5, with an additional participant sample, was an online replication of the critical E4. In E1, colored emoticons were categorized in a 5AFC task. In E2–E5, stimulus affective meaning was assessed using visual scales with anchors corresponding to each emotion. The conditions varied in stimulus arrays: E2: light gray emoticons; E3: colored circles; E4 and E5: colored emoticons. The affective meaning of Angry and Sad emoticons was found to be stronger when conferred in warm and cool colors, respectively, the pattern highly consistent between E4 and E5. The affective meaning of colored emoticons is regressed to that of achromatic expression counterparts and decontextualized color. The findings provide evidence that affective congruency of the emoticon expression and the color it is rendered in facilitates recognition of the depicted emotion, augmenting the conveyed emotional message.

## Introduction

### Emoticons

In computer-mediated communication, emoticons (a portmanteau of “emotion” and “icon”) are broadly used as pictorial “proxies” of facial expressions that serve as substitutes of nonverbal cues of real face-to-face communication (Walther & D’Addario, 2001). Emoticons help to augment the text by conveying emotions implied in the message (Tossell et al., 2012), or “playing catch with emotions,” as coined by Matsumoto (2007, p. 43). A “happy” emoticon originates from the predigital-era “smiley,” created by Harvey Ball in 1969 as a yellow button with two black dots for the eyes and a curve for the mouth. With the naissance of the Internet, this emoticon was adopted as a nonverbal means of conveying an emotional “wink,” joke, or pun. The smiley became an inspiration for the creation of emoticons varying in both expression and design (Tomić et al., 2014). Since their inception, a substantial amount of research has been dedicated to investigating (i) the role of emoticons as socio-emotional providers in messages, among other channels of written communication (for reviews, see Aldunate & González-Ibáñez, 2017; Derks et al., 2008b); (ii) the variety and frequency of emoticon use in mixed-gender communication (e.g., Tossell et al., 2012); and (iii) the adequacy of emoticon comprehension in intercultural communication (e.g., Cheng, 2017; Kavanagh, 2010; Park et al., 2013; Sugimoto & Levin, 2000; Takahashi et al., 2017).

Historically, emoticons with various expressions have been rendered in yellow – as were the original smileys intended to convey positive emotion, since yellow is associated with happiness and joy (Palmer et al., 2013) in the US culture where emoticons were designed. We are unaware of studies that systematically explored the impact of color on the semantics of the emoticon's affective message. On Google Images, we observe that as of quite recently, colored emoticons serve as vote buttons in some surveys designed for children, or therapy clients, or consumers: high satisfaction is usually coded by a yellow smiley, low satisfaction by a blue “sad” emoticon, and a “neutral” emoticon is rendered in green. Instances of other color-coding schemes are also observed, although green smileys or “sad” red or orange emoticons rarely seem to be used; “fear” and “disgust” emoticons, in general, are less common in computer-mediated communication.

Recognition of emoticons, pictorial symbols of human facial expressions, is expected to be impacted by the evinced color–emotion associations in a human face, but also by a (culture-specific) affective meaning of the color emoticons are rendered in. Below we present an overview of the findings on face color–emotion associations; this is complemented by a brief overview of emotion associations with decontextualized color and color words.

### Face Color–Emotion Associations: Biologically Engrained Face Coloration

Facial coloration conveys meaningful and valuable social information that influences interpersonal communication (Thorstenson, 2018) and is suggestive of emotional states (Thorstenson et al., 2018, 2021). A person flushes experiencing anger (Drummond, 1997) or joy (Drummond, 1994), and turns pale experiencing fear (Drummond, 1997). According to the Biopsychosocial Model of Challenge and Threat (Blascovich, 2013), an experienced emotion is accompanied by certain cardiovascular responses impacting facial skin coloration. Specifically, approach-oriented emotions (anger, happiness, surprise) evoked by challenge elicit vasodilation, facilitating blood flow to skin areas, with the face becoming redder and yellower. Conversely, avoidance-oriented emotions (disgust, fear, sadness), triggered by threat, elicit vasoconstriction, reduce blood flow to the face and, hence, incur bluer or greener facial coloration.

Several recent studies have explored associations between color and emotion expressions in images of realistic faces. Facial red was found to be judged positive as the attribute manifesting health or dominance (Re et al., 2011; Stephen et al., 2009, 2012; Thorstenson et al., 2017). Nakajima et al. (2017) systematically varied color attributes (hue, brightness, saturation) in morphed facial expression images. The authors found that perception of anger was enhanced by rendering reddish skin color, whereas perception of sadness was enhanced by blueish coloration; also, compared to morphs expressing other emotions varying in shades of gray, sad faces were more likely to be judged bluish.

Thorstenson et al. (2018), who asked participants to manipulate color in realistic face images in association with specific emotion words, found that face redness and yellowness were increased for approach-oriented emotions but decreased for avoidance-oriented emotions. Facial coloration was also found to facilitate the disambiguation of confused emotions that share facial-muscular expressive features: when the face color of realistic images was redder, these were more likely to be categorized as anger, but as disgust when rendered greener; such manipulation of color also facilitated disambiguation of two other emotion pairs, surprise–fear and happiness–sadness, respectively (Thorstenson et al., 2021).

An association of emotional expressions with color was investigated using schematic faces (Takahashi & Kawabata, 2018), the only study, to our knowledge, that explored such associations in pictorial representations of facial expressions. Participants were presented with achromatic schematic faces and instructed to “imagine the facial color” and choose a corresponding color from a palette (varying in hue, brightness, and saturation). The authors found that skin-like colors were frequent choices; *anger* was associated with saturated red, *joy* with pinkish colors and orange, *sadness* with bluish colors, and *no emotion* with white.

### Are Color–Emotion Associations Specific for a Face or Generalized to Other Stimulus Modes?

Apart from faces, associations of red with both anger and happiness, two emotions opposite in valence but high in arousal, and blue with sadness are attested for decontextualized color, patches, and geometric shapes (e.g., Suk & Irtel, 2010; Valdez & Mehrabian, 1994). Within the ecological valence theory framework, it was found that happiness is associated with vivid and pastel yellow, orange, and red (Palmer et al., 2013; Palmer & Schloss, 2010). Beyond this framework, Hanada’s (2018) conjecture that the primary association of color and emotions is mediated by perceived temperature; hence, colors that are associated with a high temperature in the environment make one experience positive emotions, i.e., feel “happy,” whereas low (cool) temperature is associated with “sadness.”

Oyama (2003) examined affective meanings of color, achromatic geometric figures, and their combinations as colored figures. He found that the affective meaning of colored figures was affected by the affective meanings of both color and achromatic figures, the approach we are pursuing in the present investigation of the interaction of color and emoticon expressions.

Thorstenson et al. (2018), who asked participants to manipulate color in abstract shapes in association with specific emotion words, found that color associations for the approach-oriented emotion terms were similar to those obtained for the face stimuli in the same study; however, for the avoidance-oriented emotions, color associations were significantly less pronounced. In other words, for the avoidance-oriented emotions, bluer and greener shifts were exacerbated in the face context. These findings could imply that (i) affective meaning of the face color, mediated by emotion terms, is projected on the affective meaning of abstract shapes, and (ii) in geometric figures, the color is associated with emotion terms in a less constraint way. The latter finding is in accord with Tan and Stephen’s (2013) report that sensitivity to color changes in the face is greater than changes in color patches.

The specific color–emotions associations delineated above, in essence, are replicated for emotion words, as demonstrated across languages and cultures: the term ‘red’ is associated with ‘anger’ and ‘love’; ‘yellow’ with ‘joy’; and ‘blue’ with ‘sadness’ (Fugate & Franco, 2019; Jonauskaite et al., 2020a; Mohr et al., 2018; Takahashi & Kawabata, 2018). The ‘blue’–‘sadness’ association can also be attributed to linguistic factors, with the “blue mood” metonymy deeply entrenched in many languages (Jonauskaite et al., 2020a; Niemeier, 1998; Takahashi & Kawabata, 2018), including the Japanese idiom *buru ni naru* ‘to become sad.’

It is argued that patterns of color–emotion associations are similar for patches and words (Jonauskaite et al., 2020b). However, other studies showed that judgment of affective meaning of color varies depending on the stimulus mode, i.e., whether a color face, color patch or color word elicits it (Takahashi & Kawabata, 2018). One is also reminded of the point made by Suk and Irtel (2010) and, more recently, by Schloss et al. (2020) that one needs to be aware of what exactly is being judged in the affective meaning of color – namely, emotions produced by sensorial processes, that reflect changes in basic physiological patterns; or linguistic convention; or “pleasantness”; or valence, i.e., color attractiveness/aversiveness derived from experiences with color objects in the world. The crucial role of the stimulus mode and the attribute judged can be illustrated by the following example: unlike ‘sad’ association with the ‘blue’ term, decontextualized blue was judged “pleasant”; in comparison, while ‘yellow’ term is associated with ‘joy,’ decontextualized yellow patch was found to be the “least pleasant” among other colors (Schloss et al., 2020; Shirai & Soshi, 2021; Suk & Irtel, 2010; Valdez & Mehrabian, 1994). In addition, the sign of the emotion instilled by color is greatly affected by lightness and saturation: vivid blue was judged “happy” whereas dark and pale yellow “sad” (Schloss et al., 2020).

### Factors Modulating Color–Emotion Associations in Pictorial Face Representations

These bearings may be relevant in the context of color variation in artificial “faces” of emoticons: along with associations between human face coloration and emotion, stipulated by biologically engrained predispositions (“nature”), the symbolic, anthropomorphized emoticon expressions can potentially evoke affective responses to their color that reflect repeated life experiences, social conventions and culture-specific semiotics (“nurture”). For example, red judged positive as the face attribute manifesting health or dominance can evoke negative associations implicated by conventional signs of danger or failure, exemplifying color symbolism (Elliot et al., 2007; Oyama, 2003).

The affective meaning of color can also be culture-specific. Adams and Osgood (1973), who compared eight color concepts in 23 cultures, concluded that there exist “universals” in how people feel about color, with particularly salient concepts of ‘red’ and ‘blue,’ both with highly positive affective value. However, divergences from the universal tendencies were found in some cultures, too, attributed to culture-specific symbolism of the color. Within the Osgood framework of the primary factors of the affective meaning (Activity, Evaluation, Potency), Oyama et al. (1962) compared the affective value of color patches by Japanese and US participants and found that “cultural contamination” was most powerful in the evaluative judgments of colors.

### Present Study

Although numerous studies examined the association between emotion and facial color, as well as decontextualized colors, geometric shapes, and color words, to our knowledge, the impact of color variation on emotions read out from emoticons has not been addressed. In the present study, we explored how color variation affects recognition of emotions that emoticons are intended to convey. We predicted that the color of emoticon would affect the semantics of its affective message, in particular, that emoticons conveying negative emotions would be read out optimally when rendered in colors other than yellow. If this indeed is the case, unknowingly to the sender's intention, conventional yellow “sad” or “angry” emoticons may trigger an “emotional Stroop” effect, whereby the conventional yellow color would bias the receiver's reading of the affective meaning of “negative” emoticons.

We report four lab-based experiments (E1-E4), where the task and/or the stimulus array was varied by exploring accuracy and response speed of emotion recognition, as well as the affective meaning attributed to the emoticons as a function of color. Further, upon a reviewer's suggestion, in E5, we replicated the critical E4 with a different participant sample that had not completed E1-E3 experiments; the additional experiment was carried out online (in lockdown circumstances).

We hypothesized that (i) congruency between the affective meaning of the emoticon expression and the color it is rendered in would facilitate recognition of the conveyed emotion; conversely, semantically incongruent color–emotion combinations would attenuate recognition of the emoticon's message; (ii) affective meaning of the colored emoticon can be predicted from the affective meaning of the achromatic emoticon and the affective meaning of decontextualized color.

## Method

### Participants

In Experiments 1–4 (E1–E4), participants were young females (N = 22) aged 18–22 years (M = 19.4 ± 0.8) enrolled for studies at a Japanese private female-only university. All had normal color vision as assessed by Ishihara pseudoisochromatic plates (Ishihara, 2008). Each participant was tested individually and completed E1–E4 on the same day. The experiments were run in a fixed order, from E1 through E4, with a total duration of about 1.5 h, including short breaks. Before the experiments, participants were dark adapted for 10 min; they were seated 60 cm in front of the monitor in an otherwise dark room. The study was approved by the Ethical Committee of Joshibi University of Art and Design, Tokyo. All participants provided the informed consent.

In the online Experiment (E5), we tested a different group of participants (N = 30), aged 19–23 years (M = 20.3 ± 1.0), who were recruited from both Kanagawa University (N = 22; 12 males, all majoring in Human Sciences) and Joshibi University of Art and Design (N = 8; all females). The participants were sent the link to the experimental program and carried out the experiment under natural daylight illumination at a location they were comfortable in, with the total duration of about 70 min. Their color vision was tested online prior to the experiment using an online proxy of the Ishihara test (https://enchroma.com/pages/test?clk_src = top_menu2). The participants were required to report the message (“diagnosis”) yielded upon the test completion. In all cases “normal color vision” was fed back. E5 was approved by the Ethical Committees of Kanagawa University and Joshibi University of Art and Design. All participants provided the informed consent.

### Stimuli

In E1–E4, stimuli were presented on a 24.1” LCD Monitor (EIZO ||ColorEdge CS2420, 1920 × 1200 pixels). Stimulus color was measured using a SpectraScan PR-650 (Photo Research Inc.) colorimeter. The stimuli were presented on a light gray background (CIE *x* = 0.287, *y* = 0.314, *Y* = 81.70). Seven chromatic colors were best possible approximations, in both chromaticity and luminance, of the corresponding colors (predominantly of the ‘Saturated’ subset) in the Berkeley Color Project (see Table S1 in Palmer & Schloss, 2010). The eighth color in the present study was Light Gray identical to that of the background (Figures S1, S2 in Supplementary Materials). Circular stimuli constituted 4° × 4° of visual angle. The experimental program was run in Visual Basic (Balena & Fawcette, 1999).

In E5, the stimuli were presented on personal mobile devices (iPads or cell phones). The stimulus color was specified in the sRGB color space, with the coordinates being the best possible approximation of the RGB values of the *xyY*-coordinates of the colors used in the lab-based experiment (Table S1 in Supplementary Materials). The visual angle of the stimulus apparently varied between participants due to the size of the mobile device display and, also, was smaller than in the lab condition.

**Figure 1.**
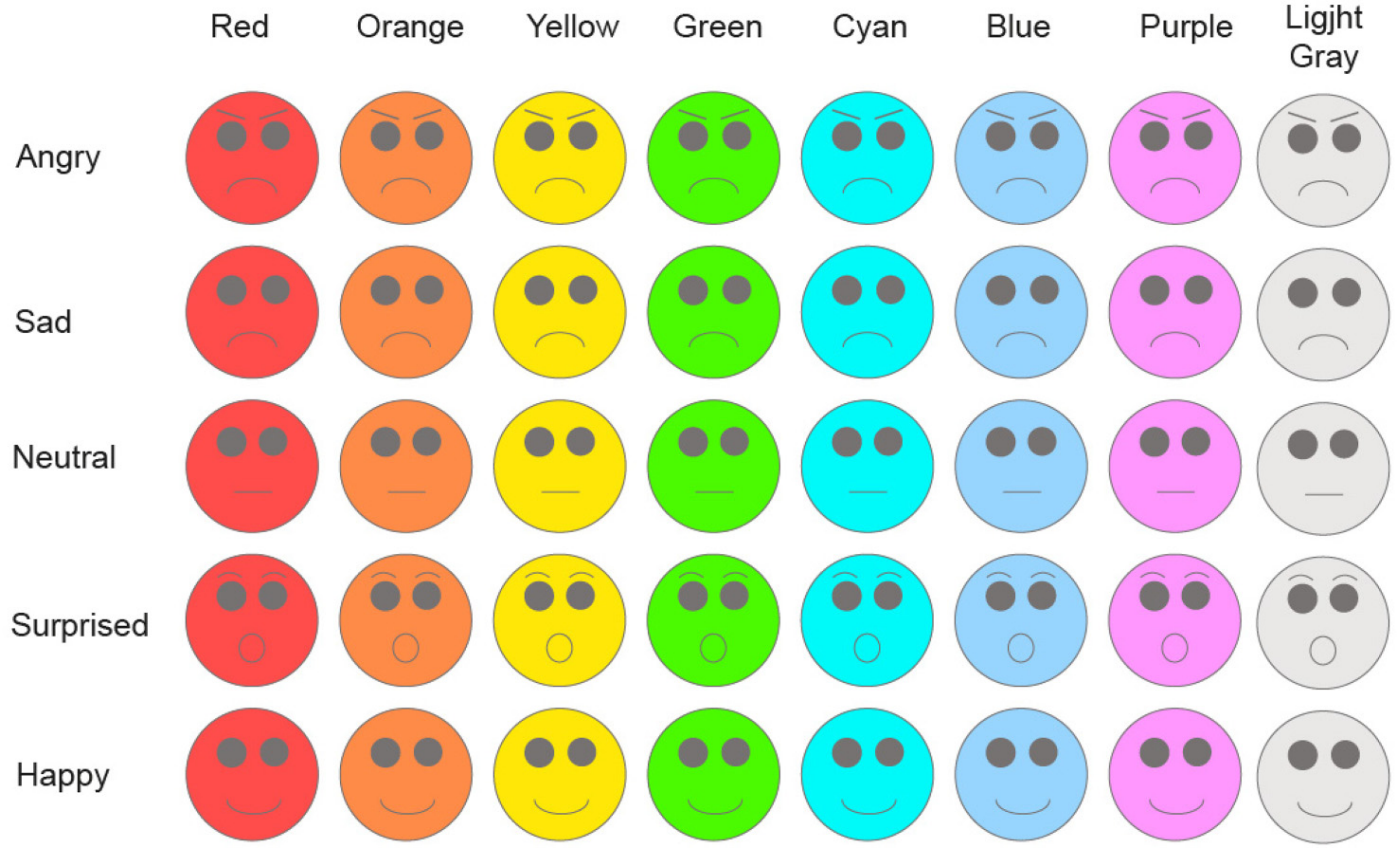
Forty emoticons (5 basic emotions × 8 colors) employed in the present study.

In both lab-based and online experiments, stimuli were presented in the screen center as singletons in random order. In E1, E2, E4, and E5, the stimulus sets consisted of emoticons representing four basic emotions – Angry, Sad, Surprised, and Happy, from the six emotions of the Ekman and Friesen (1971) inventory and a Neutral emoticon. (Depictions of the other two basic emotions, fear, and disgust, were considered less common in computer-mediated communication.) In E1, E4, and E5, each emoticon was presented in eight colors (Figure 1). In E2, only Light Gray emoticons were presented. The contour and “features” of the emoticons were rendered in dark gray. (An initial stimulus design revealed that rendered in black, the eye dots appeared hollow. We are grateful to Stephen Palmer for pointing out the “hole in the face” impression and his advice on the dark gray color of the features.). In E3, circles of the same eight colors were presented.

## Procedure

### Experiment 1: Categorization of Emotions in Emoticons Varying in Color

Underneath the emoticon, five virtual buttons were aligned horizontally and labeled as “Angry,” “Sad,” “Neutral,” “Surprised,” “Happy” in a fixed order, from left to right. (For the Japanese terms used and English glosses, see Table S2). The task of the participants was to mouse-click, as quickly and accurately as possible, on the button that best reflected their impression of the emotion in the emoticon. The stimulus was presented until the response was provided. The response was followed by a 500 ms interval to prevent an afterimage effect. Each of the five emoticons was presented in eight colors 5 times, with the total number of trials N = 5 emoticons× 8 colors× 5 = 200.

### Experiment 2. Assessing Affective Meaning of Achromatic Emoticons

In E2, we explored whether the emoticon's affective meaning was conveyed as intended in the absence of any chromatic color. The stimulus set was composed of Light Gray emoticons depicting the four emotions and the neutral expression. Participants assessed the affective meaning of each emoticon on five unipolar affective meaning (AM) visual scales with the following anchors (in Japanese; see Table S2): “Not Angry–Angry,” “Not Sad–Sad,” “Not Neutral–Neutral,” “Not Surprised–Surprised,” and “Not Happy–Happy.” The participant's task was to assess the emoticon AM by mouse-clicking on the visual scale placed beneath the emoticon; at the start of each trial, the black marker was located in the middle of the scale. The presentation of the five emoticons was randomized, as was the presentation of the AM scales for the emoticons. The mouse-click was followed by a 500 ms interval, when this expired presentation of the next emoticon was triggered, accompanied by one of the AM scales. (This presentation timeline was also implemented in E3 and E4.) Each emoticon was presented twice, with each of the AM scales, resulting in 50 trials (N = 5 emoticons× 5 AM scales× 2).

### Experiment 3. Assessing Affective Meaning of Decontextualized Color

Participants were presented with eight colored circles and assessed the affective meaning of each of the colors on the five AM scales identical to those in E2. Each colored circle was presented randomly, twice, accompanied by the presentation of the five AM scales, also presented randomly, resulting in 80 trials in total (N = 8 colors × 5 AM scales × 2).

### Experiment 4. Assessing Affective Meaning of Colored Emoticons

**Figure 2.**
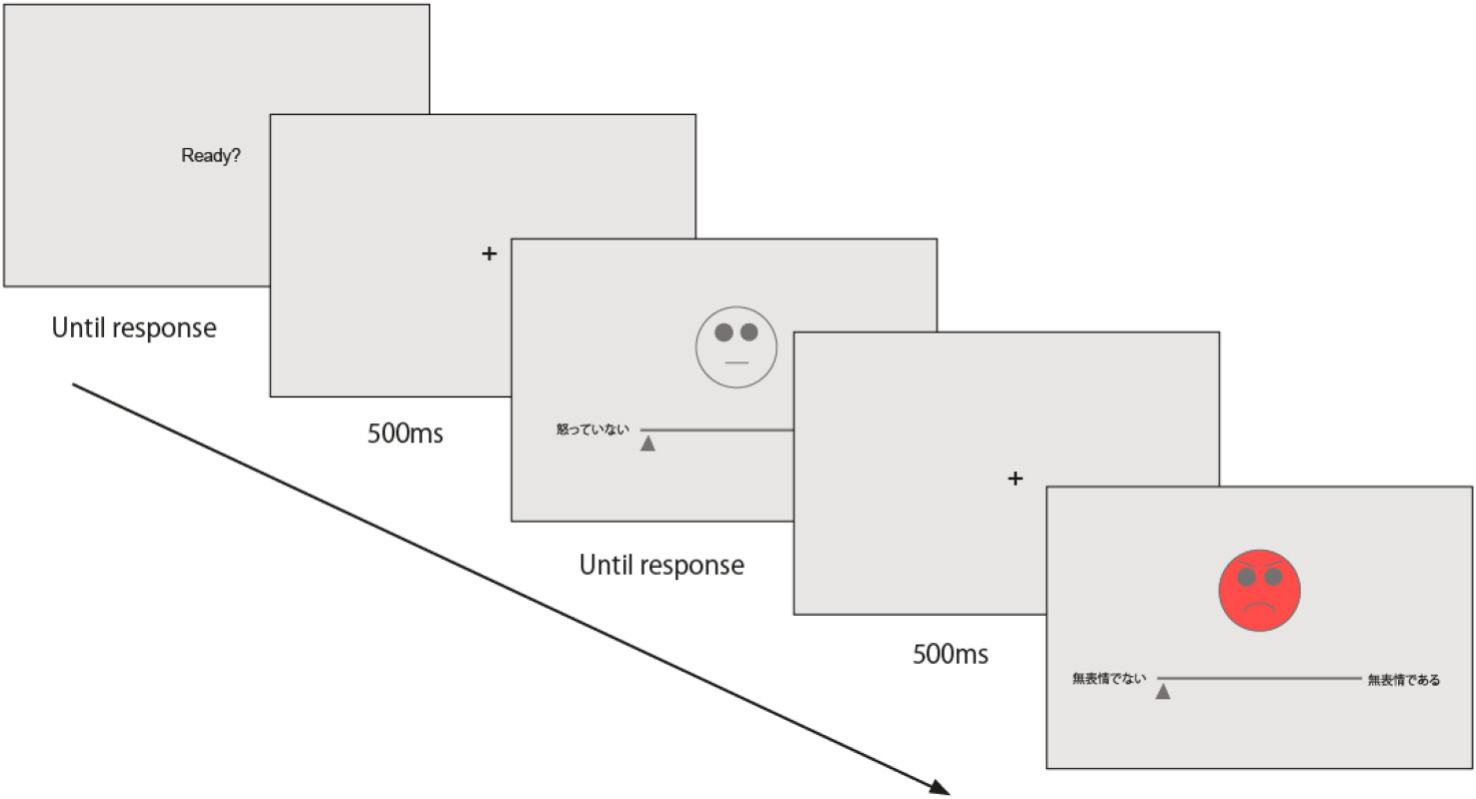
An illustration of the procedure in Experiments 4 and 5.

In the critical E4, we tested the affective meaning of each colored emoticon. Participants evaluated the affective meaning of all emoticons on each of the five AM scales (see Figure 2), same as in E2 and E3. Each emoticon–color combination was presented two times resulting in 400 trials in total (N = 5 emoticons × 8 colors × 5 AM scales × 2).

### Experiment 5. Assessing Affective Meaning of Colored Emoticons: An Online Replication

E5 was an online replication of E4, whereby participants assessed the affective meaning of colored emotions (see Figure 2). In E5, each emoticon–color combination was presented two times, too, resulting in 400 trials in total (N = 5 emoticons × 8 colors × 5 AM scales × 2). E5 was carried out in response to reviewers’ concerns of the possible “carry-over effect” in E4: since all participants have completed E1-E4, in a fixed order and on the same day, in E4, they might have cognitively adapted their responses to the purpose of the experiment inferred from the stimuli and tasks in E1-E3.

### Behavioral Measures and Data Analysis

In E1, the corrected responses to the emoticon were counted (varying between 0–5); RTs (ms) were recorded, too, and means of correct RTs were calculated. In E2–E5, for analysis of the AM of the stimuli, i.e., emoticons (E2, E4, E5) and colored circles (E3), a magnitude estimation method was applied (Ehrenstein & Ehrenstein, 1999). The position of the participant's mouse clicks (E2, E4; desktop computer) or dragging (E5; mobile device) on the AM scale was transformed into numerical values between 0 (left) and 100 (right).

For each experiment, a one-way repeated-measures ANOVA (analysis of variance) of AM estimates was conducted to test possible main effects of color separately on each emoticon. Where assumptions of sphericity were violated, corrected degrees of freedom are indicated. In *post hoc* analysis conducted for pairwise comparisons provided that a main effect was found, the Bonferroni correction was used. In addition, a linear regression analysis was undertaken to test the prediction that affective meaning of colored emoticons (E4) could be inferred from the affective meaning of the colored circles (E3) and the affective meaning of Light Gray emoticons (E2) with the corresponding expression, the approach inspired by the study of colored geometric figures by Oyama (2003). Outcomes of E4 and E5 were related using Pearson's correlation analysis. ANOVA was conducted using SPSS 26.0; regression and correlation analyses were run on Microsoft Excel 16.56.

## Results

### Experiment 1: Categorization of Colored Emoticons

**Figure 3.**
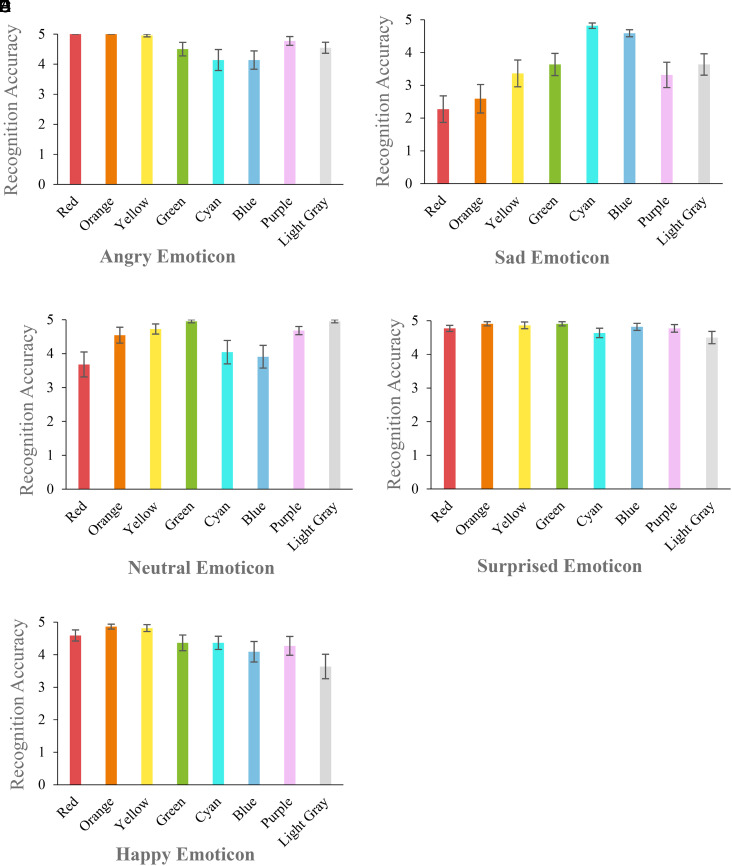
Experiment 1: profiles of categorization accuracy (means and standard errors) of Angry (A), Sad (B), Neutral (C), Surprised (D), and Happy (E) emoticons rendered in the eight colors.

The overall accuracy rate was 87.6%, which indicates that generally, the emotion in the colored emoticons was recognized as intended, regardless of the color in which they were rendered. Profiles of the categorization accuracy for each of the five emoticons varying in color are presented in Figure 3.

Outcomes of ANOVA indicated a significant main effect of color on accuracy of categorization of Sad [*F*(3.357, 70.507) = 16.612, *MSE* = 2.107, *p* < .001, η^2^ = .442], Neutral [*F*(2.674, 56.145) = 7.206, *MSE* = 1.934, *p* = .001, η^2^ = .255], and Happy emoticons [*F*(3.025, 63.525) = 5.491, *MSE* = 1.482, *p* = .002, η^2^ = .207]. There was no significant main effect of color on RTs.

*Post hoc* analysis showed that for ***Sad emoticon*** (Figure 3B), accuracy [assessed by mean and standard error (SE)] was highest for Cyan (M = 4.82 ± 0.09) and Blue (M = 4.59 ± 0.11) compared to all other colors (all *p*s < .05); the accuracy was rather low in Red (M = 2.27 ± 0.41) and Orange (M = 2.59 ± 0.43).

For ***Neutral emoticon*** (Figure 3C), accuracy was highest when it was rendered in Light Gray (M = 4.95 ± 0.05) and Green (M = 4.95 ± 0.05); it was significantly lower when rendered in Red (M = 3.68 ± 0.37; *p*s ≤ .05).

***Happy emoticon*** (Figure 3E), as expected, revealed higher categorization accuracy when rendered in Orange (M = 4.86 ± 0.07) and Yellow (M = 4.82 ± 0.11), both higher than in Light Gray rendering (M = 3.64 ± 0.38; *p*s ≤ .05).

### Experiment 2: Affective Meaning of Achromatic Emoticons

**Figure 4.**
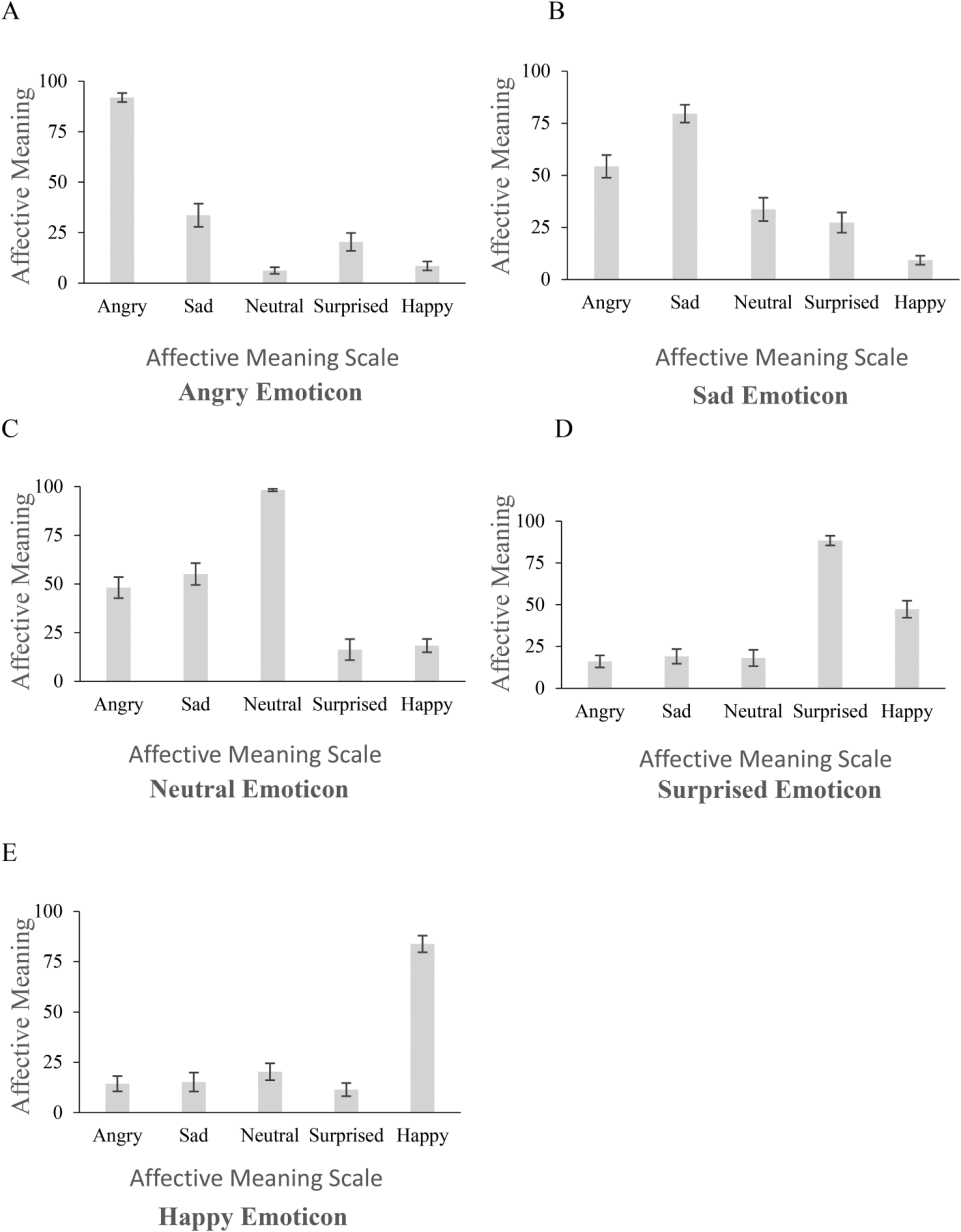
Experiment 2: profiles (means and standard errors) of the affective meaning (AM) estimates of Light Gray emoticons on the five AM scales.

The intended emotions were appropriately recognized in all five Light Gray emoticons. Figure 4 shows that means of AM values on the scale corresponding to the conveyed emotion were close to 100 or at least 80, definitely higher than those on the other four AM scales (see Table S3).

ANOVA outcomes of estimates of Light Gray emoticons show that across the five AM scales, the AM estimates significantly differed for each emoticon with large effect size (all *p*s < .001; see Table S3). Estimates of the emotion intended in the emoticon were much higher than those on other AM scales confirmed by outcomes of *post hoc* analysis of pairwise differences.

***Angry*** and ***Happy emoticons*** were read out rather unmistakably: they had the highest Angry AM and Happy AM estimates, respectively, than on other AM scales (all *p*s < .001).

Each of the three other emoticons elicited at least one secondary affective meaning. In particular, ***Sad emoticon*** appeared to convey an Angry ‘residual’ emotion: Sad AM was higher than Angry (*p* = .008) and other AM values (all *p*s < .001); in turn, Angry AM was higher than Surprised and Happy AM values (*p*s < .001).

In comparison, ***Surprised emoticon*** revealed a relatively high Happy AM estimate, i.e., instilled a positive emotion; its Happy AM was higher than Angry (*p* = .002), Sad (*p* = .001), or Neutral AM (*p* = .015).

***Neutral emoticon***, along with the Neutral AM, revealed negative ‘residuals’: its Angry and Sad AM values were higher than Surprised (*p*s < .001) and Happy AM (*p*s ≤ .004).

### Experiment 3: Affective Meaning of Color Circles

**Figure 5.**
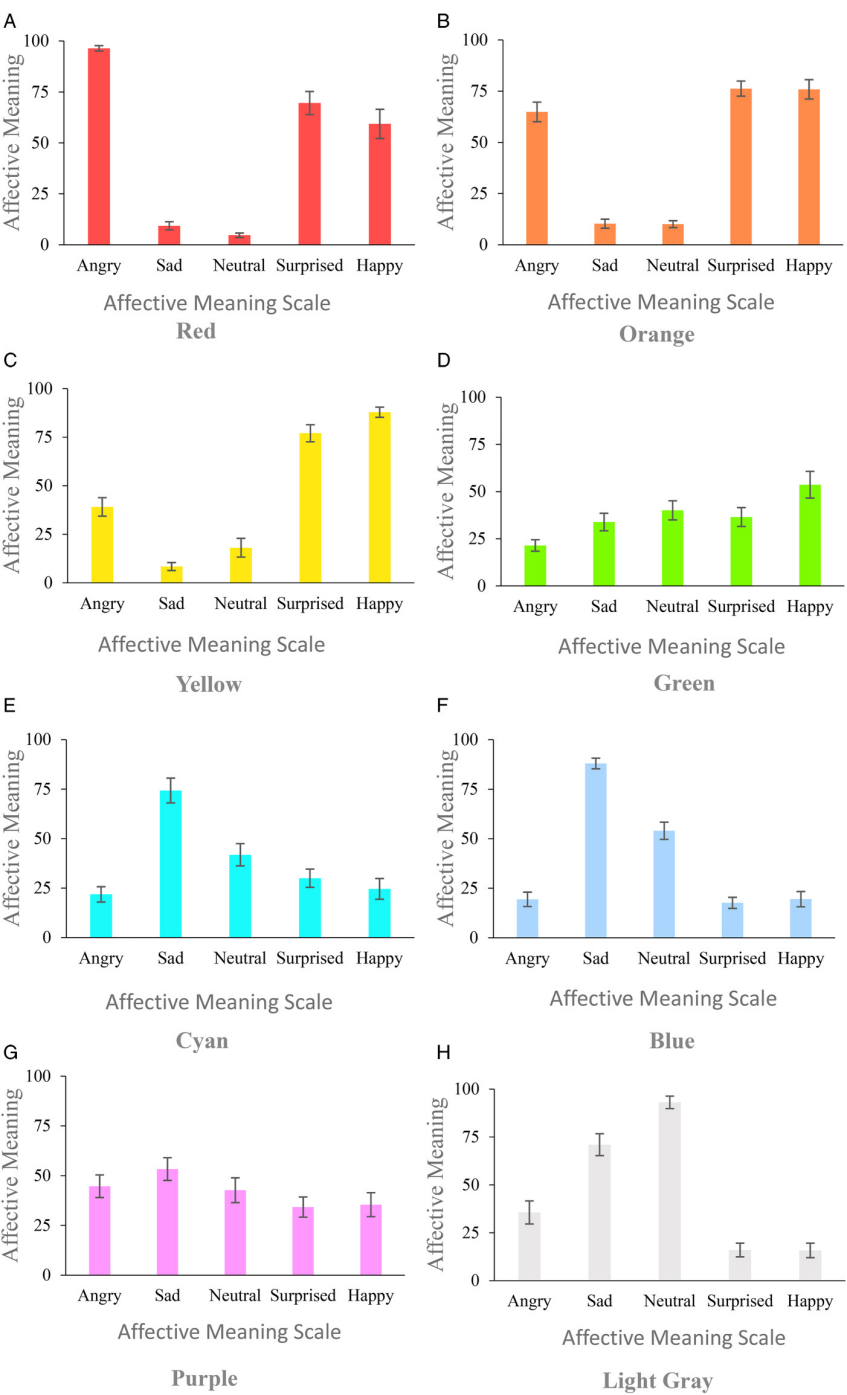
Experiment 3: profiles of affective meaning estimates (means and standard errors) of the eight colors on the five AM scales.

Figure 5 shows AM estimates of the eight colors on the five AM scales. ANOVA confirmed a significant main effect of emotion (reflected by the AM scale value) in relation to each decontextualized color, except Purple. [Means (SEs) of AM estimates and ANOVA outcomes for individual colors are presented in Table S4.]

***Red*** and ***Orange*** (Figure 5A, B) share a similar affective profile in that both colors instigate high Angry AM but also high Happy and Surprised AM. Red had higher Angry values than on other AM scales (all *p*s < .001), followed by Surprised and Happy AM, with both higher than Sad and Neutral (all *p*s < .001).

For ***Yellow*** (Figure 5C), Happy and Surprised AM estimates were the highest (all *p*s < .001).

***Green*** (Figure 5D) instilled moderate Happy and Surprised AMs, higher than Angry AM (*p* = .008 and *p* = .030, respectively).

***Cyan*** and ***Blue*** (Figure 5E, F) are similar in their affective profiles, too, sharing the highest Sad AM. For Cyan, Sad AM was higher than Angry, Neutral, Surprised, and Happy (all *p*s ≤ .004). For Blue, Sad AM was higher than values on all other AM scales (all *p*s < .001).

***Light Gray*** (Figure 5H) had the highest Neutral AM estimate, greater than Sad (*p* = .018), Angry, Surprised, and Happy (all *p*s < .001); it also instilled a rather high Sad AM, greater than Surprised and Happy AM (*p*s < .001).

In comparison to other colors, ***Purple*** (Figure 5G) did not reveal any distinctive AM profile; although Sad AM was slightly higher it did not differ significantly from values on other AM scales.

### Experiment 4: Affective Meaning of Colored Emoticons

**Figure 6.**
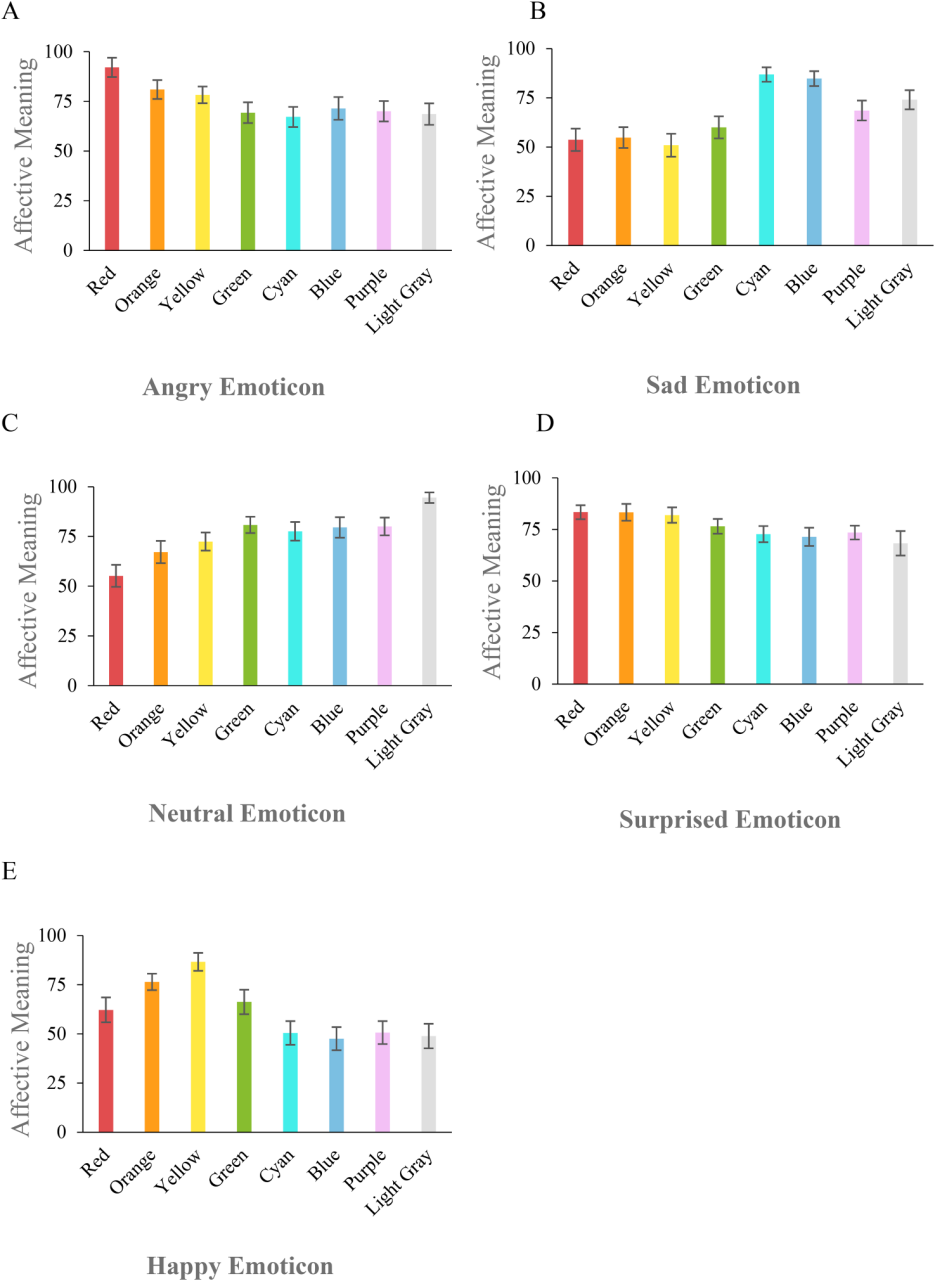
Experiment 4: profiles of affective meaning estimates (means and standard errors) of the emoticons rendered in the eight colors on the AM scale corresponding to the emotion implicated in the emoticon. Note. Estimates of (A) Angry emoticon on the “Not Angry–Angry” scale; (B) Sad emoticon on the “Not Sad–Sad” scale; (C) Neutral emoticon on the “Not Neutral–Neutral” scale; (D) Surprised emoticon on the “Not Surprised–Surprised” scale; (E) Happy emoticon on the “Not Happy–Happy” scale.

Figure 6 shows participants’ AM judgments of emoticons rendered in each of the eight colors on the AM scale that corresponds to the emotion implicated in the emoticon. Means (SEs) for the emoticon in each color on each of the five AM scales and ANOVA outcomes are presented in Table S5 of Supplementary Materials. ANOVA showed a main effect of color on the reading of emotion in each colored emoticon across the five AM scales (all *p*s < .001).

As is apparent, ***Angry emoticon*** (Figure 6A) was perceived as “angrier” in warm colors, particularly in ***Red*** and ***Orange***. Red Angry emoticon had higher Angry AM than in all other colors (all *p*s < .05), and Orange Angry emoticon was “angrier” than in Light Gray (*p* = .031).

***Happy emoticon*** (Figure 6E), as expected, was judged “happiest” in ***Yellow***: the corresponding AM was higher than when rendered in Purple and Light Gray (*p*s < .001) or Red, Cyan, and Blue (all *p*s < .05). Happy emoticon was also judged rather “happy” in ***Orange***, with Happy AM higher than when it was rendered in Cyan, Blue, Purple, or Light Gray (all *p*s < .05). In Green, Happy AM was greater than in Light Gray (*p* = .044).

Conversely, ***Sad emoticon*** (Figure 6B) was perceived as “sadder” in cool colors, ***Cyan*** and ***Blue***: for both colors, Sad AM was higher than when in Red, Orange, Yellow, or Green (all *p*s < .001). It is worth noting that ***Purple*** Sad emoticon evinced Sad AM estimate that was only marginally lower than when rendered in either Cyan (*p* = .058) or Blue (*p* = .081).

***Neutral emoticon*** was judged as most “neutral” in ***Light Gray*** (Figure 6C), with the corresponding AM significantly higher than in Red (*p* < .001), Orange, or Yellow (all *p*s < .05).

Finally, ***Surprised emoticon*** (Figure 6D) appeared to slightly better convey the intended emotion when rendered in ***Red***, higher than in Blue (*p* = .037), in accord with findings for realistic face images (Thorstenson et al., 2021).

### Affective Meaning of the Colored Emoticons: Impact of the Expression in Achromatic Emoticon and the Decontextualized Color

A multiple linear regression analysis was carried out to explore whether AM of a colored emoticon (E4) with a certain expression can be predicted from the affective meaning of the achromatic, Light Gray emoticon with the same expression (E2) and the affective meaning of the corresponding colored circle (E3). Mean values on each AM scale for the five emoticons (E2) and the seven chromatic colors (E3) were used as the predictors, while values on the five AM scales for all colored emoticons (E4) were treated as the outcome variable (N = 770 = 5 emotions × 7 colors × 22 participants). Note that the AM values for Light Gray color circles (E3) and Light Gray emoticons (E4) were excluded from the analysis not to duplicate these conditions’ data as both predictor and outcome variables.

The following linear regression equations were obtained for colored emoticons with the specific emotion expression, where CE, E, and C represent AMs of the colored emoticon, of the corresponding expression in the achromatic emotion, and of the corresponding decontextualized color, respectively:

***Angry*** (CE) = 0.66(E) + 0.34(C); *F*(2, 770) = 490.87, *p* < .001, *R*^2^ = .56

***Sad*** (CE) = 0.53(E) + 0.47(C); *F*(2, 770) = 420.82, *p* < .001, *R*^2^ = .52

***Neutral*** (CE) = 0.68(E) + 0.32(C); *F*(2, 770) = 253.12, *p* < .001, *R*^2^ = .40

***Surprised*** (CE) = 0.74(E) + 0.26(C); *F*(2, 770) = 651.31, *p* < .001, *R*^2^ = .63

***Happy*** (CE) = 0.75(E) + 0.25(C); *F*(2, 770) = 601.71, *p* < .001, *R*^2^ = .61

The regression equations provide evidence that the affective meaning of the colored emoticon is significantly predicted by the affective meaning of both the expression of the counterpart achromatic emoticon and the decontextualized color. The AMs of the colored emoticons appear to be close to a weighted average of the AM values of the emotion expression and the color; we therefore constrained the sum of regression coefficients to 1. However, and not unexpectedly, the impact of the depicted emotion is greater than that of the color. Interestingly, for Angry, Sad, and Neutral emoticons, the regression coefficients of the color are relatively higher than those for Surprised and Happy emoticons prompting that color may have a greater impact on recognition of non-positive expressions.

### Experiment 5: Affective Meaning of Colored Emoticons: An Online Study Replication

The pattern of results in E5 (see Figure S3) was greatly consistent with the pattern obtained in E4 (cf. Table S5 and Table S6). ANOVA of E5 data, conducted for each emoticon, showed a main effect of the color (*p*s ≤ .001) and no effect of gender or expertise.

***Angry emoticons*** were judged “angrier” when rendered in Red and Orange. For ***Red*** emoticon, Angry AM was higher than in Cyan, Light Gray (all *p*s < .001) or in Yellow, Green, Blue and Purple (all *p*s < .05). For ***Orange*** emoticon, Angry AM was higher than in Cyan (*p* = .003).

***Sad emoticon*** in ***Blue*** and ***Cyan***, as in E4, had higher Sad AM than in all other colors (all *p*s < .05).

***Neutral emoticon*** was judged as “no emotion” expression prevalently in ***Light Gray***, compared to when it was rendered in other colors: Red and Orange (*p*s < .001), Yellow, Green, Cyan, Blue, and Purple (all *p*s < .05).

***Surprised emoticon*** judgments in E5 deviated, however, from those in E4, with its AM profile across the colors being similar to that of Happy emoticon: Surprised AM was highest in ***Yellow*** and ***Orange*** and, as well, relatively high in ***Purple***, all higher than in Blue (all *p*s ≤ .020).

***Happy emoticon*** was “happier” in ***Yellow, Orange***, and, unlike E4, in ***Purple***; in these three colors, Happy AM was higher than in Red, Green, Cyan, Blue, or Light Gray (all *p*s < .05).

In E5, the affective impact of ***Purple*** appears to be similar to that of warm colors (cf. Table S5 and Table S6). An independent sample *t-*test showed that Sad emoticon in Purple was less “sad” than in E4 [*t*(50) = −3.75, *p* < .001], while Happy emoticon was “happier” than in E4 [*t*(50) = 3.82, *p* < .001], hinting that in E5 Purple was rendered more reddish than it was in E4.

Finally, we related the outcomes of E4 and E5 to address the possible “carry-over effect” in E4. Pearson correlation coefficients were computed between the values in both experiments, for each emoticon colored in each on eight colors and on each AM scale (see Figure S4 of Supplementary Materials). We found strong positive correlations, with large effect sizes, for Angry [*r*(6) = .95, *p* < .001, *R^2^* = .90], Sad [*r*(6) = .85, *p* = .007, *R^2^* = .72], and Neutral emoticons [*r*(6) = .90, *p* = .002, *R^2^* = .81], between the E4 and E5 values on the AM scale corresponding to the emotion intended in the emoticon.

The correlations were medium and marginally significant for Surprised [*r*(6) = .62, *p* = .098] and Happy emoticons [*r*(6) = .63, *p* = .094]. The values were probably lower due to the discrepancy in E4–E5 AM estimates of these emoticons when rendered in Purple. To test this assumption, we rerun correlation analysis for these two emoticons by excluding AM estimates for Purple rendering. The so obtained correlation coefficients raised indeed: for Surprised emoticon [*r*(5) = .73, *p* = .063, *R^2^* = .53], and for Happy emoticon [*r*(5) = .98, *p* < .001, *R^2^* = .95]. The still marginally significant correlation for Surprised emoticon hints at factors at play other than color, which we contemplate upon in the Discussion.

In general, the strong positive correlations between the outcomes in the two independent experiments suggest that a “carry-over effect” was unlikely in judgments of affective meaning of colored emoticons in E4 due to participants’ exposure to the stimuli in E1-E3.

## Discussion

The present study demonstrates that the decoding of the intended emotion from emoticons does indeed depend on the color in which the emoticon is rendered, as reflected by the attributed affective meaning. Along with the expression of the implicated emotion, affective meaning of colored emoticon is impacted by the affective meaning of the color it is rendered in. Or, in statistical terms, affective meaning of colored emoticon can be regressed to the affective meaning of achromatic emoticons and the decontextualized color, although the latter plays a lesser role. The impact of both characteristics for colored emoticons is in accord with Oyama’s (2003) finding that affective meaning of colored geometric figures is regressed to those of both colors and achromatic figures.

### Congruency Effect of the Emotion Conveyed by an Emoticon and of the Color Affective Meaning

The present findings provide evidence of a congruency effect, i.e., an addition of the affective meaning of the emoticon expression and the color it is rendered in, whereby emoticons in congruent colors are decoded more correctly. Specifically, Happy emoticons (smileys) are perceived as “happier” in conventional yellow or orange; Angry emoticons convey the intended emotion stronger when presented in red; Sad emoticons are “sadder” when rendered in blue or cyan; and Neutral emoticons best convey their non-emotional message when rendered in gray.

In contrast, Angry emoticons in cool colors and Sad and Neutral emoticons in warm colors revealed higher chances of being misinterpreted. These findings indicate that incongruent emotion expression–color combinations attenuate the message conveyed by the emoticons. The resulting ambiguity of a message apparently triggered an “emotional Stroop” effect that potentially could lead to a receiver's misinterpretation of the context, the tone of the message, or the sender's attitude – be it a negative bias, flaming the interaction, or a positive bias with spurious peace-making – but ultimately impacting the efficiency of communication (cf. Derks et al., 2008a; Sugimoto & Levin, 2000; Walther & D’Addario, 2001). The present findings can be useful in developing communication tools in social networking sites to improve the “emotion catch-ball” in digital communications (Matsumoto, 2007, p. 43) by using emoticons with congruent color variations instead of conventional yellow (unicolor) emoticons. As boldly put by Chaudhuri (n.d., p. 13), “[t]oo many factors can interfere with the bare message and mislead its final meaning. In Japan, an emoticon can be a life-saver.”

### The Impact of Color on Emoticons’ Reading is Drawn from Both Expression Semantics and Real Face Color

The results of the present study, demonstrating the impact of color in emoticons, are in accord with the findings in studies on associations of the emotional expression and facial coloration in realistic images (Minami et al., 2018; Nakajima et al., 2017; Thorstenson et al., 2018). Note, though, that in realistic face images, the variation of color chromaticity is both more constrained and subtle than that in emoticons. For example, in Thorstenson et al.'s (2018) study, participants varied the color within ± 20 along both a* and b* axes (in CIELAB space), when asked to manipulate facial coloration, in realistic face images and the color of abstract shapes, in association with the perceived emotion. In comparison, in the present study, the colors of the stimuli varied within a much larger range [a* *=* (–71)–55; b* *=* (–34)–83], which is much less realistic than face colors experienced in real-life communication.

Nevertheless, the patterns of the expression–color associations that are similar for both realistic face images and emoticons indicate that the semantics of face color (stipulated in physiological and psychological models of emotion) is translated to the semantics of emoticon color. Furthermore, the “exaggerated” colors of emoticons were tolerated by observers and, in terms of the affective meaning, instigated expected associations, provided that the chromaticity of the emoticon's non-natural color did not change the sign, on the a* (red-green) and b* (blue-yellow) components in color space (cf. Figure S2) in relation to the associated natural face color. This prompts that in artificial “faces,” the color is likely to acquire symbolic meaning.

### Colors Manifesting a Lesser Effect on Emoticons’ Reading are Hardly Present in Real Face Coloration

In the present color set, Green and Purple were included to enable comparison with outcomes of the Berkeley Color Project with decontextualized color patches. Our results prompted, however, that these two colors are ambiguous and barely helpful in facilitating the emoticon affective message since they are unlike shades of coloration of a human face in everyday life: they rarely emerge in some extreme conditions, such as capillary fragility, which results in high hemoglobin concentration manifested by purplish-colored skin, or very low blood oxygen, whereby face color may acquire a greenish taint (Changizi et al., 2006). Noteworthy, decontextualized Purple did not produce specific affective responses in E3 (Figure 5). In comparison, in E4, the Sad AM of Sad emoticon in Purple was only marginally lower than when it was rendered in the “diagnostic” Cyan and Blue, i.e., cool colors (see Figure 6B; Table S5).

### Emoticons Conveying Negative Emotions are More Strongly Affected by the Color Variation

We showed that congruency between the color affective meaning and the emotion conveyed by the emoticons, as reflected by a higher recognition accuracy and higher attributed affective meaning, was most pronounced for Angry and Sad emoticons, i.e., those conveying negative emotions. This is also confirmed by greater regression coefficients of the color (C), estimated for individual colored emoticons, i.e., 0.34(C) for Angry and 0.47(C) for Sad emoticons, compared to 0.25(C) for Happy and 0.26(C) for Surprised emoticons. Noteworthy, for Neutral emoticon, the regression coefficient of color is also relatively high, 0.32(C), which is likely to be explained by the finding for realistic neutral faces that neutral expressions contain predominantly negative “emotion residue” (Albohn & Adams, 2020; Lee et al., 2008).

From an evolutionary viewpoint, the finding of the greater impact of color in judging negative expressions may be explained by the significance of fast and effective extracting of threat-related cues from the face to enhance awareness of negative emotions in a communication partner and orchestrate appropriate behavioral responses (Öhman et al., 2001; Vuilleumier, 2002). Apparently, this face-to-face communication phenomenon can be extended to symbolized digital communication in the social network environment.

The marked impact of color on emoticons conveying negative emotions can also be related to the specific participant population in the present study, since a display of negative emotions is discouraged in the Japanese culture (Hutchison et al., 2018; Matsumoto, 1992, 2007; Sugimoto & Levin, 2000). As a consequence, the Japanese are more anxious in interpersonal contexts and are more vigilant and sensitive to signs of disapproval (Dailey et al., 2010; Ishii et al., 2011).

### The Emotion Conveyed by an Emoticon Appears to Override the Affective Meaning Impacted by Color Lightness

In the present study, we employed the colors that well represented the corresponding categories and with the highest saturation achieved on the employed monitor; this ensued that the colors varied in lightness (see Table S1). Several previous studies that explored color–emotion associations using color patches demonstrated that the nature of the association depends not so much on hue but predominantly on lightness and chroma (saturation) (Elliot & Maier, 2012; Jonauskaite et al., 2020b; Suk & Irtel, 2010; Valdez & Mehrabian, 1994). In a recent study, where color–emotion association was systematically explored under the variation of all three color attributes, Schloss et al. (2020) found that red–angry association is dominated by the hue; in comparison, the associations of yellow with happiness and of blue with sadness change their affective sign depending on the lightness and chroma variation: dark and desaturated yellow hues were not rated as “happy”; conversely, light and saturated blue hues were.

In the current study, Happy emoticons rendered in a moderately light Orange (*Y* = 43 cd/m^2^) were perceived as “happier” than Happy emoticons rendered in light Cyan (*Y* = 83 cd/m^2^), suggesting that, unlike decontextualized color patches, in emoticons, the expressed emotion in combination with the “optimal” color override the lightness effect, the proposition that is testable.

### Possible Cross-Cultural Differences in Recognition of Colored Emoticons

We are cognizant that generalization of the present findings of the effect of color on the decoding of emoticon's affective meaning requires a study comparing the performance of observers from Eastern and Western cultures. To address this question, we also collected data in the UK, using similar stimulus colors, and identical emoticons and experimental protocol (Thorstenson, Schloss, & Paramei, in preparation). A preliminary analysis indicates cross-cultural similarities in reading colored emoticons (Liao et al., 2019), which echoes Ou et al.'s (2004) conclusion that participants with significant differences in language and geography (in their study, British and Chinese) revealed consensus on most of the color–emotion associations.

In the present study with Japanese observers, we also observe some performance outcomes, in both recognition of achromatic emoticons and color–emotion associations, that may be culture-specific. We imply, in particular, cases of misattributions of affective meaning of achromatic emoticons (E2): Sad emoticons were ascribed Angry AM and vice versa, while Surprised emoticons obtained relatively high Happy AM estimates (Figure 4).

The Angry–Sad confusion may be related to the fact that the Japanese experience greater difficulty (than US Americans) in recognizing negative facial expressions unless the negative emotion is “fully expressed” (cf. Shioiri et al., 1999), the finding attributed to discouraging the display of negative emotions in the Japanese culture (Matsumoto, 1992, 2007).

These Angry–Sad and Surprised–Happy confusions may also be explained by a specific facial expression decoding strategy of Japanese observers: unlike Western viewers, whose judgments of facial expressions are dominated by the shape of the mouth, Japanese observers (and those in other Eastern cultures) are more likely to judge facial emotions from the eyes (Gedron, 2017; Hutchison et al., 2018; Jack et al., 2009; Sugimoto & Levin, 2000; Yuki et al., 2007) or eyebrows (Hasegawa & Unuma, 2010). This decoding strategy, or the “own emotional dialect” (Dailey et al., 2010, p. 875), is reflected in the design of conventional Japanese emoticons: the mouth is represented by a straight line, regardless of the conveyed emotion, whereas the emotion valence is conveyed by the eye shape, with capital lambdas standing for “happy eyes” (^_^) and capital Ts for “sad eyes” (T_T) (Park et al., 2013; Takahashi et al., 2017). The round eye shape (0_0) symbolizes a positive emotion of curiosity and amazement (Park et al., 2013), which may have triggered relatively high “Happy” estimates of Surprised emoticon.

In relation to the latter finding, one can speculate that a lower (and marginally significant) E4–E5 correlation of AM estimates of Surprised emoticon was caused by a smaller size of the stimuli in E5, where smaller mobile device displays were used compared to the lab-based E4. The emotion inferred from a face subtending a smaller visual angle is determined by low spatial-frequency (SF) “diagnostic” features, while in larger face stimuli, higher SFs are processed too (Smith & Schyns, 2009). In line with this argument, in E5, the emotion inferred from Surprised emoticon was dominated by the shape of the mouth and eyes, whereby for Japanese observers, the prominent round eyes probably enhanced the emoticon perception as “happily surprised.” In comparison, in E4, with larger face stimuli, the eye–eyebrow distance had an impact, too, on the reading of this emoticon's expression. Since in our design of Surprised emoticon, the eye–eyebrow distance was relatively small, it probably resembled the “diagnostic” feature (lowered eyebrows) of an angry expression, in E4 shifting the affective meaning of Surprised emoticon to “angrily surprised.” This consideration can be tested empirically; if confirmed, it implies that for optimally conveying emotions by “icons” on social media devices, the design of emoticons requires taking into account “the diagnostic SF spectrum” of the emotion expression (Smith & Schyns, 2009, p. 1203).

### Concluding Remarks

The findings of the present study showed a consistent color effect on recognition of emoticon expressions, which can be useful for developing communication means on social networking sites. In the meantime, a more comprehensive cross-cultural investigation would be advisable to enable culture-specified modifications in intercultural communication (Cheng, 2017; Kavanagh, 2010). Since young adults exercise computer-mediated communication intensely, this might have resulted in a more fluent and effective interpretation of the (colored) emoticons compared to the middle-aged or older who are less experienced in this regard. Disentangling potential impacts of the degree of digital literacy on reading the affective meaning of (colored) emoticons and decontextualized colors is worth exploring too.

The online experiment E5 also provided us with an opportunity to obtain participants’ judgments of affective meaning of colored emoticons in a simulated online communication environment, with participants viewing the stimuli on their portable devices. We were aware of the downside of an online experiment, such as less control over the display and viewing conditions, and the stimulus color, and the subsequent increase in variation of individual judgments. However, we were encouraged by a recent study by Hirao et al. (2021), who compared the perception of face obtained online and under controlled lab conditions. The authors conclude that results in the tasks performed online were broadly consistent with lab-based results, provided that a participant sample is sufficiently large to overcome a statistical error.

## Supplemental Material

sj-docx-1-ipe-10.1177_20416695221080778 - Supplemental material for Color Affects Recognition of Emoticon Expressions Click here for additional data file. Supplemental material, sj-docx-1-ipe-10.1177_20416695221080778 for Color Affects Recognition of Emoticon Expressions by Songyang Liao, Katsuaki Sakata and Galina V. Paramei in i-Perception
