# Supplementary material for: Color Affects Recognition of Emoticon Expressions
Source: Iperception. 2022 Feb 28;13(1):20416695221080778. doi: 10.1177/20416695221080778 (PMC8900290; doi:10.1177/20416695221080778)
Supplement: sj-docx-1-ipe-10.1177_20416695221080778 - Supplemental material for Color Affects Recognition of Emoticon Expressions [file sj-docx-1-ipe-10.1177_20416695221080778.docx]

Supplementary Materials

**Figure S1**

*Eight Colors used in the Present Study ( ) and the Corresponding Subset of the Berkeley Color Project Colors ( ) (Palmer & Schloss, 2010) Plotted in the xy-Plane of the CIE 1931 Chromaticity Diagram*

**Figure S2**

*Projections of the Eight Colors onto the Chromatic a*b*-Plane of the CIELAB space, where the a*-Axis represents the Opponent Red- (+a*) –Green- (-a*) components, and the b*-Axis Represents the Opponent Yellow- (+b*) –Blue- (-b*) Components of Color.*

**Figure S3**

*Experiment 5: Profiles of Affective Meaning (AM) Estimates (Means and Standard Errors)* *of the Emoticons Rendered in the Eight Colors on the AM Scale Corresponding to the Emotion Intended in the Emoticon*

**Figure S4**

*(A) Correlations of AM Estimates on the AM Scale Corresponding to the Emotion Intended in the Emoticon between the E4 (Lab-Based) and E5. (B) Correlations of AM Estimates for Surprised and Happy Emoticons when Purple was Excluded from the Analysis.*

B

**Table S1**

CIE 1931 Coordinates (E1-E4) and sRGB (E5) of the Emoticon Colors in the Present Study. For Comparison Shown are CIE 1931 Coordinates of Similar Colors in the Berkeley Color Project (Palmer & Schloss, 2010).

|  | Present study | | | | | |  | Berkeley Color Project | | |
| --- | --- | --- | --- | --- | --- | --- | --- | --- | --- | --- |
| **Color** | *x* | *y* | *Y* (cd/m^2^) | *R* | *G* | *B* |  | *x* | *y* | *Y* (cd/m^2^) |
| Red | 0.51 | 0.34 | 33.78 | 255 | 77 | 76 |  | 0.55 | 0.31 | 22.93 |
| Orange | 0.49 | 0.39 | 43.42 | 255 | 139 | 72 |  | 0.51 | 0.41 | 49.95 |
| Yellow | 0.43 | 0.47 | 84.46 | 255 | 232 | 8 |  | 0.45 | 0.47 | 91.25 |
| Green | 0.32 | 0.55 | 71.53 | 75 | 250 | 0 |  | 0.39 | 0.50 | 68.56 |
| Cyan | 0.22 | 0.31 | 82.53 | 0 | 249 | 249 |  | 0.23 | 0.34 | 49.95 |
| Blue | 0.23 | 0.29 | 65.79 | 151 | 213 | 255 |  | 0.26 | 0.28 | 59.25 |
| Purple | 0.28 | 0.23 | 55.60 | 255 | 151 | 255 |  | 0.29 | 0.24 | 49.95 |
| Light Gray | 0.29 | 0.31 | 81.70 | 234 | 231 | 231 |  | 0.31 | 0.32 | 63.90 |
| Dark Gray (emoticon contour & “features”) | 0.27 | 0.30 | 16.82 | 120 | 113 | 113 |  | 0.31 | 0.32 | 19.26 |

**Table S2**

Japanese Characters for the Terms of the Five Basic Emotions and the Anchor Labels of the Affective Meaning Scales Accompanied by English Glosses

| Japanese characters | English glosses |
| --- | --- |
| 怒り | Angry |
| 悲しみ | Sad |
| 無表情 | Neutral |
| 驚き | Surprised |
| 喜び | Happy |
| 怒っていない—怒っている | Not-Angry—Angry |
| 悲しくない—悲しい | Not-Sad—Sad |
| 無表情でない—無表情である | Not-Neutral—Neutral |
| 驚いていない—驚いている | Not-Surprised—Surprised |
| 喜んでいない—喜んでいる | Not-Happy—Happy |

**Table S3**

*Experiment 2: Light Gray Emoticons. Estimates of the Affective Meaning (AM) on the Five AM Scales [Mean (M) and Standard Error (SE)], (the Emotion Implied in the Emoticon is Highlighted in Bold), and Outcomes of the Repeated-Measures ANOVA*

|  | **Angry** AM | | **Sad** AM | | **Neutral** AM | | **Surprised** AM | | **Happy** AM | |
| --- | --- | --- | --- | --- | --- | --- | --- | --- | --- | --- |
| **Emoticon** | M | SE | M | SE | M | SE | M | SE | M | SE |
| Angry | **91.91** | **2.22** | 33.64 | 5.76 | 6.23 | 1.60 | 20.42 | 4.40 | 8.55 | 2.22 |
| Sad | 54.30 | 5.45 | **79.58** | **4.24** | 33.61 | 5.60 | 27.32 | 4.85 | 9.25 | 2.17 |
| Neutral | 48.11 | 5.40 | 55.09 | 5.60 | **98.18** | **0.62** | 16.30 | 5.40 | 18.32 | 3.44 |
| Surprised | 16.02 | 3.59 | 19.07 | 4.38 | 18.07 | 4.87 | **88.40** | **2.90** | 47.30 | 5.07 |
| Happy | 14.36 | 3.81 | 15.18 | 4.72 | 20.30 | 4.23 | 11.41 | 3.28 | **83.83** | **4.15** |
|  | *df* | | *F* | | MSE | | η² | | *p* | |
| Angry | 2.398, 50.363 | | 98.844 | | 458.473 | | .825 | | <.001 | |
| Sad | 4, 84 | | 39.747 | | 16031.320 | | .654 | | <.001 | |
| Neutral | 2.520, 52.923 | | 69.431 | | 559.689 | | .768 | | <.001 | |
| Surprised | 2.343, 49.201 | | 55.171 | | 657.777 | | .724 | | <.001 | |
| Happy | 1.852, 38.885 | | 79.175 | | 569.783 | | .790 | | <.001 | |

**Table S4**

*Experiment 3: Color Circles. Estimates of the Affective Meaning (AM) of the Eight Colors on the Five AM Scales [Mean (M) and Standard Error (SE)] and Outcomes of the* *Repeated-Measures ANOVA*

|  | **Angry** AM | | **Sad** AM | | **Neutral** AM | | **Surprised** AM | | **Happy** AM | |
| --- | --- | --- | --- | --- | --- | --- | --- | --- | --- | --- |
| **Color** | *M* | *SE* | *M* | *SE* | *M* | *SE* | *M* | *SE* | *M* | *SE* |
| Red | **96.41** | **1.32** | 9.30 | 2.01 | 4.68 | 1.08 | **69.55** | **5.70** | **59.32** | **7.18** |
| Orange | **64.85** | **4.78** | 10.30 | 2.22 | 10.09 | 1.69 | **76.27** | **3.70** | **75.90** | **4.75** |
| Yellow | 39.10 | 4.79 | 8.39 | 2.06 | 18.08 | 4.84 | **77.05** | **4.41** | **87.85** | **2.63** |
| Green | 21.41 | 3.05 | 33.85 | 4.67 | 40.05 | 5.08 | 36.49 | 5.00 | **53.60** | **7.09** |
| Cyan | 21.84 | 3.84 | **74.30** | **6.28** | 41.85 | 5.63 | 30.00 | 4.62 | 24.59 | 5.22 |
| Blue | 19.42 | 3.63 | **87.98** | **2.70** | 54.02 | 4.37 | 17.63 | 2.81 | 19.50 | 3.85 |
| Light Gray | 35.63 | 6.01 | **70.97** | **5.72** | **93.10** | **3.25** | 16.00 | 3.61 | 15.78 | 3.81 |
|  | *df* | | *F* | | MSE | | η² | | *p* | |
| Red | 2.117, 44.448 | | 95.123 | | 689.517 | | .819 | | <.001 | |
| Orange | 2.644, 55.518 | | 92.689 | | 423.676 | | .815 | | <.001 | |
| Yellow | 4, 84 | | 83.729 | | 325.635 | | .799 | | <.001 | |
| Green | 2.489, 52.264 | | 4.731 | | 1005.477 | | .184 | | .008 | |
| Cyan | 2.338, 49.100 | | 15.657 | | 1103.275 | | .427 | | <.001 | |
| Blue | 4, 84 | | 90.893 | | 232.508 | | .812 | | <.001 | |
| Purple | 2.787, 58.525 | | 1.900 | | 994.021 | | .083 | | .143 | |
| Light Gray | 4, 84 | | 57.677 | | 454.111 | | .733 | | <.001 | |

**Table S5**

*Experiment 4: Colored Emoticons. Estimates of the Affective Meaning (AM) of Each Emoticon Rendered in the Eight Colors on the AM Scale Corresponding to the Emotion Implicated in the Emoticon [Mean (M) and Standard Error (SE)], and Outcomes of the Repeated-Measures ANOVA. Highlighted in Bold are the Color(s) that Elicited Highest AM Estimates*

| **Emoticon** | **Red** | | | **Orange** | | **Yellow** | | **Green** | | **Cyan** | | **Blue** | | **Purple** | | **Light Gray** | |
| --- | --- | --- | --- | --- | --- | --- | --- | --- | --- | --- | --- | --- | --- | --- | --- | --- | --- |
|  | *M* | | *SE* | *M* | *SE* | *M* | *SE* | *M* | *SE* | *M* | *SE* | *M* | *SE* | *M* | *SE* | *M* | *SE* |
| Angry | **92.16** | 4.85 | | **80.99** | 4.76 | 78.28 | 4.21 | 68.28 | 5.22 | 67.16 | 5.06 | 71.45 | 5.74 | 70.00 | 5.14 | 68.61 | 5.40 |
| Sad | 53.72 | 5.67 | | 54.82 | 5.31 | 50.94 | 5.83 | 60.03 | 5.62 | **86.85** | 3.69 | **84.80** | 3.76 | 68.57 | 5.06 | 74.06 | 4.85 |
| Neutral | 55.18 | 5.52 | | 67.14 | 5.59 | 72.42 | 4.58 | 80.81 | 4.08 | 77.55 | 4.69 | 79.52 | 5.12 | 80.00 | 4.47 | **94.49** | 2.68 |
| Surprised | **83.36** | 3.36 | | **83.30** | 4.08 | **81.98** | 3.73 | 76.51 | 3.59 | 72.74 | 3.91 | 71.41 | 4.40 | 73.49 | 3.34 | 68.26 | 5.91 |
| Happy | 62.20 | 6.30 | | **76.45** | 4.16 | **86.66** | 4.60 | 66.27 | 6.23 | 50.48 | 6.00 | 47.58 | 5.87 | 50.66 | 5.81 | 48.90 | 6.25 |
|  | *df* | | | | | *F* | | | MSE | | | η² | | | *p* | | |
| Angry | 3.448, 72.412 | | | | | 8.602 | | | 380.521 | | | .291 | | | <.001 | | |
| Sad | 4.284, 89.969 | | | | | 15.593 | | | 458.952 | | | .426 | | | <.001 | | |
| Neutral | 7, 147 | | | | | 10.185 | | | 284.008 | | | .327 | | | <.001 | | |
| Surprised | 4.167, 87.501 | | | | | 4.852 | | | 262.809 | | | .187 | | | <.001 | | |
| Happy | 3.936, 82.661 | | | | | 13.123 | | | 626.305 | | | .385 | | | <.001 | | |

**Table S6**

*Experiment 5: Colored Emoticons, Online Replication. Estimates of the Affective Meaning (AM) of Each Emoticon Rendered in the Eight Colors on the AM Scale Corresponding to the Emotion Implicated in the Emoticon [Mean (M) and Standard Error (SE)], and Outcomes of Repeated-Measures ANOVA. Highlighted in Bold are the Color(s) that Elicited Highest AM Estimates*

| **Emoticon** | **Red** | | **Orange** | | **Yellow** | | **Green** | | **Cyan** | | **Blue** | | **Purple** | | **Light Gray** | |
| --- | --- | --- | --- | --- | --- | --- | --- | --- | --- | --- | --- | --- | --- | --- | --- | --- |
|  | *M* | *SE* | *M* | *SE* | *M* | *SE* | *M* | *SE* | *M* | *SE* | *M* | *SE* | *M* | *SE* | *M* | *SE* |
| Angry | **92.09** | 2.73 | **83.64** | 2.93 | 78.35 | 2.47 | 76.89 | 2.17 | 74.83 | 2.93 | 77.04 | 2.97 | **79.06** | 3.34 | 76.47 | 2.95 |
| Sad | 52.78 | 3.72 | 58.81 | 3.87 | 58.42 | 3.78 | 58.33 | 4.19 | **72.83** | 3.59 | **73.72** | 3.99 | 53.67 | 4.73 | 61.67 | 4.43 |
| Neutral | 66.84 | 4.31 | 75.59 | 3.03 | 80.42 | 3.80 | 81.13 | 2.75 | 76.06 | 3.51 | 79.28 | 3.68 | 76.11 | 3.62 | **93.70** | 2.73 |
| Surprised | **75.05** | 2.66 | **77.90** | 2.62 | **81.15** | 3.37 | 70.35 | 3.95 | 71.94 | 2.70 | 65.44 | 3.56 | **77.59** | 2.44 | 72.24 | 3.44 |
| Happy | 69.56 | 3.86 | **79.39** | 2.97 | **81.11** | 3.38 | 71.16 | 3.69 | 65.07 | 3.64 | 65.24 | 3.61 | **82.21** | 2.89 | 66.16 | 4.44 |
|  | *df* | | | | *F* | | | MSE | | | η² | | | *p* | | |
| Angry | 4.462, 129.391 | | | | 7.828 | | | 189.234 | | | .213 | | | <.001 | | |
| Sad | 4.671, 137.020 | | | | 9.407 | | | 316.888 | | | .245 | | | <.001 | | |
| Neutral | 4.518, 131.026 | | | | 8.823 | | | 299.818 | | | .233 | | | <.001 | | |
| Surprised | 4.758, 137.980 | | | | 4.827 | | | 227.534 | | | .143 | | | .001 | | |
| Happy | 4.937, 143.169 | | | | 8.273 | | | 275.045 | | | .222 | | | <.001 | | |
